# Hepatopulmonary syndrome: an update

**DOI:** 10.1590/S1516-31802009000400008

**Published:** 2009-12-07

**Authors:** Liana Gonçalves de Macêdo, Edmundo Pessoa de Almeida Lopes

**Affiliations:** 1 MD, MSc. Pulmonologist at the Otávio de Freitas Hospital, Recife, Pernambuco, Brazil.; 2 MD, PhD. Adjunct professor at the Health Sciences Center, Universidade Federal de Pernambuco (UFPE), Recife, Pernambuco, Brazil.

**Keywords:** Hepatopulmonary syndrome, Liver transplantation, Liver cirrhosis, Hypertension, portal, Echocardiography, Liver cirrhosis, experimental, Síndrome hepatopulmonar, Transplante de fígado, Cirrose hepática, Hipertensão portal, Ecocardiografia, Cirrose hepática experimental

## Abstract

Hepatopulmonary syndrome (HPS) is a clinical threesome composed of liver disease, intrapulmonary vascular dilatation (IPVD) and arterial gas abnormalities. Its occurrence has been described in up to 32% of cirrhotic candidates for liver transplantation. It also affects non-cirrhotic patients with portal hypertension. Its pathogenesis is not well defined, but an association of factors such as imbalance in the endothelin receptor response, pulmonary microvascular remodeling and genetic predisposition is thought to lead to IPVD. Diagnosis is based on imaging methods that identify these dilatations, such as contrast echocardiography or perfusion scintigraphy with ^99m^Tc, as well as analysis of arterial gases to identify elevated alveolar-arterial differences in O_2_ or hypoxemia. There is no effective pharmacological treatment and complete resolution only occurs through liver transplantation. The importance of diagnosing HPS lies in prioritizing transplant candidates, since presence of HPS is associated with worse prognosis. The aim of this paper was to review the pathogenetic theories and current diagnostic criteria regarding HPS, and to critically analyze the prioritization of patients with HPS on the liver transplant waiting list. Searches were carried out in the Medline (Medical Literature Analysis and Retrieval System Online) via PubMed, Cochrane Library and Lilacs (Literatura Latino-Americana e do Caribe em Ciências da Saúde) databases for articles published between January 2002 and December 2007 involving adults and written either in English or in Portuguese, using the term *hepatopulmonary syndrome*. The studies of greatest relevance were included in the review, along with text books and articles cited in references that were obtained through the review.

## RESUMO

A síndrome hepatopulmonar (SHP) é considerada uma tríade clínica composta de doença hepática, dilatações vasculares intrapulmonares (IPVD) e alterações de gases arteriais. Há descrição de sua ocorrência em até 32% dos cirróticos candidatos ao transplante de fígado, acometendo também não cirróticos com hipertensão portal. Sua etiopatogenia não está bem definida, mas se especula que a associação de fatores como o desequilíbrio na resposta dos receptores vasculares de endotelina, o remodelamento microvascular pulmonar e a predisposição genética propiciem as IPVD. O diagnóstico baseia-se em métodos de imagem que identifiquem essas dilatações, como a ecocardiografia com contraste ou a cintilografia de perfusão com ^99m^Tc, além da análise de gases arteriais, para identificar elevação da diferença alvéolo-arterial de O_2_ ou hipoxemia. Não existe tratamento medicamentoso eficaz e sua resolução completa ocorre apenas com o transplante de fígado. A importância do diagnóstico da SHP está em priorizar os candidatos ao transplante, uma vez que sua presença está associada ao pior prognóstico. O objetivo deste artigo é fazer revisão das teorias de etiopatogenia da SHP, dos seus critérios diagnósticos atuais, além de realizar análise crítica sobre a prioridade dos pacientes com a síndrome na lista de espera pelo transplante de fígado. Buscaram-se na base de dados Medline (Medical Literature Analysis and Retrieval System Online) via PubMed, Cochrane Library e Lilacs (Literatura Latino-Americana e do Caribe em Ciências da Saúde), os artigos publicados no período de janeiro de 2002 a dezembro de 2007, envolvendo adultos, escritos nos idiomas inglês ou português, que apresentassem o termo síndrome hepatopulmonar. Os estudos de maior relevância foram incluídos, além de livros-texto e artigos citados nas referências obtidas na revisão.

## INTRODUCTION

The first publication to describe an association between lung disease and liver disease dates back to 1884, with a report on the coexistence of cirrhosis, cyanosis and finger clubbing in an adult patient.^1^ However, the term hepatopulmonary syndrome (HPS) was used for the first time in 1977 when the concept of intrapulmonary vascular dilatation (IPVD) was introduced.^2^ IPVD is a condition that causes gas exchange abnormalities.^3^^,^^4^

HPS is a clinical threesome composed of liver disease associated with the presence of IPVD and arterial gas abnormalities.^5^ More recently, portal hypertension has been implicated in the development of HPS, regardless of the impairment of liver function.^6^

While its pathogenesis not yet completely clear,^7^ HPS is widely prevalent (4% to 32%) among cirrhotic candidates for liver transplant.^8^^,^^9^^,^^10^^,^^11^^,^^12^ It has been associated with worse prognosis,^13^^,^^14^ and liver transplantation is the only possibility for complete reversal.^5^

The aim of the present paper was to carry out a literature review on HPS, describing recent theories on its pathogenesis, assessing current diagnostic criteria and performing a critical analysis regarding prioritizing patients with HPS on the waiting list for liver transplants. Searches were carried out with the term hepatopulmonary syndrome in the Medline (Medical Literature Analysis and Retrieval System Online) via PubMed, Cochrane Library and Lilacs (Literatura Latino-Americana e do Caribe em Ciências da Saúde) databases for articles published between January 2002 and December 2007 (Table 1). The studies considered most relevant were discussed. Textbooks and articles cited in the references that were obtained in the review were also consulted.

**Table 1.** Search strategy for “hepatopulmonary syndrome” and results in the Medline (Medical Literature Analysis and Retrieval System Online) via PubMed, Cochrane Library and Lilacs (Literatura Latino-Americana e do Caribe em Ciências da Saúde) databases, from 2002 to 2007

Databases	Search strategy	Results	n
PubMed	Hepatopulmonary syndrome [MeSH] Limits: - Publication date: January 1, 2002, to December 31, 2007 - Languages: English, Portuguese - Ages: adults (19 + years)	Case report	32
		Case series	13
		Prevalence study	9
		Case-control study	9
		Cohort study	12
		Clinical trial	2
		Experimental study	2
		Comments/Letter to editor	2
		Total	81
Cochrane Library	Hepatopulmonary syndrome [MeSH] Limits: - Publication date: 2002 to 2007	Case report	9
		Case series	2
		Prevalence study	3
		Case-control study	1
		Cohort study	5
		Experimental study	1
		Narrative review	4
		Comments/Letter to editor	3
		Total	28
Lilacs	Síndrome hepatopulmonar [DeCS] Limits: - Publication date: January 1, 2002, to December 31, 2007 - Ages: adults	Case report	1
		Prevalence study	1
		Case-control study	1
		Experimental study	1
		Narrative review	2
		Systematic review	2
		Total	8

MeSH = Medical Subject Headings; DeCS = Descritores em Ciências da Saúde.

## CHRONIC LIVER DISEASE AND LIVER TRANSPLANTATION

Liver transplantation is generally indicated as the treatment for chronic, advanced, irreversible liver disease with complications such as ascites or encephalopathy that lead to shorter life expectancy than would be observed with a transplant.^15^^,^^16^^,^^17^ In February 2002, new waiting list positioning criteria began to be used in the United States, using a score based on liver disease severity, named the Model for End-Stage Liver Disease (MELD).^18^^,^^19^ This new system has ideal model characteristics, since it is based on few parameters (all of them objective): the bilirubin and creatinine levels in blood and the prothrombin time (international normalized ratio). These parameters are easily obtainable and reproducible and provide a score standard with an excellent capability for prognoses regarding the risk of death among candidates for liver transplantation.^20^ The advantage of this system over the previous system, which used the duration of the wait for the transplant (chronological criterion), lies in the fact that it reduces the mortality rate by prioritizing urgent cases.^21^

In Brazil, until the middle of 2006, for patients to be included in the waiting list for liver transplants, they needed to present complications due to cirrhosis.^16^^,^^17^ The position on the list was according to the date of inclusion. Since then, through an ordinance issued by the Brazilian Ministry of Health, the MELD system has replaced this chronological criterion and now the position on the list is in accordance with the severity of the liver disease.^22^

In cases of liver disease with an indication for transplantation, vascular disorders with a wide variety of clinical manifestations may develop as a result of hepatocellular dysfunction or portal hypertension. These play a role in the assessment and follow-up of liver transplant candidates.^17^^,^^23^ Such disorders include gastroesophageal varices, ascites, hepatic encephalopathy, hepatorenal syndrome, portopulmonary hypertension and HPS.^24^^,^^25^

Among the diagnostic guidelines and protocols relating to HPS, liver disease was firstly included as a prerequisite for its development, but without establishing whether the liver disease would be acute or chronic.^5^ The criteria in these guidelines regarding the underlying liver disease were subsequently redirected, to suggest that portal hypertension should be present, either with or without cirrhosis.^6^ However, in most studies on HPS, it was found to develop in candidates for liver transplantation, i.e. patients with advanced liver disease, and nearly all of them were cirrhotic.^9^^,^^10^^,^^11^

The main consequence of cirrhosis is portal hypertension, which may also be caused by an increase in resistance at the pre-sinusoidal level or an increase in portal flow, as in cases of portal vein thrombosis, congenital liver fibrosis and hepatosplenic schistosomiasis.^25^^,^^26^^,^^27^

Confirming the possibility that IPVD might develop in non-cirrhotic patients with portal hypertension, HPS has been diagnosed in this population, although with a lower occurrence rate than among patients with cirrhosis.^28^^,^^29^^,^^30^ HPS has also been described in non-cirrhotic patients with chronic viral hepatitis and with normal portal pressure,^31^ as well as during the evolution of severe acute liver failure.^32^

## PATHOGENESIS OF HPS

Although not yet completely elucidated, the pathogenesis of HPS is believed to involve a number of factors. Together, these factors lead to IPVD and oxygenation abnormalities in patients with liver disease.^5^^,^^6^^,^^33^ There have been reports of genetic predisposition associated with possible imbalance in the receptors that regulate pulmonary vascular tonus.^1^ More recently, there have been reports on the role of bacterial translocation^6^ and probable remodeling in the pulmonary vascular bed.^34^^,^^35^

Just over a decade ago, there was discussion of the idea that imbalance in the synthesis of vasodilator and vasoconstrictor substances probably acted on the lungs and led to the development of IPVD, but there was a lack of experimental models to prove this.^1^ Currently, the hypotheses for explaining the pathogenesis of HPS remain speculative, despite the various experimental studies that have been conducted.^36^^,^^37^^,^^38^^,^^39^^,^^40^

Emphasis has been given to the roles that endothelin (ET-1) may play in the pulmonary microcirculation and in the genesis of IPVD.^41^ Under normal conditions, ET-1 produced in the liver has the function of regulating pulmonary vascular tonus.^25^ Upon binding to receptors located in the vascular smooth muscle tissue (ET_A_), ET-1 produces vasoconstriction. However, when binding to receptors located in the pulmonary vascular endothelium (ET_B_), it produces vasodilatation due to the synthesis of nitric oxide (NO) through stimulation of endothelial nitric oxide synthase (eNOS).^40^ Thus, ET-1 balances its vasoconstrictor effect and helps to keep the pulmonary ventilation/perfusion ratio within normal parameters.^41^ Following liver damage, ET-1 produced in the liver arrives in the pulmonary circulation and appears to preferentially interact with the ET_B_ receptor, thereby promoting pulmonary vasodilatation.^38^

In cases of liver cirrhosis, the levels of tumor necrosis factor-alpha (TNF-a) also rise, and this contributes towards the accumulation of macrophages in the lumen of the pulmonary vessels. In turn, these macrophages stimulate another NO-producing enzyme (induced nitric oxide synthase, iNOS), thereby triggering pulmonary vasodilatation.^42^ Analysis on a model for liver cirrhosis and hyperdynamic circulation following common bile duct ligation (CBDL) has revealed greater hepatic production and plasma circulation of ET-1, which is associated with the development of HPS.^39^

One study examined two groups with regard to the development of HPS. One group consisted of rats with cirrhosis following CBDL and the other consisted of rats without cirrhosis and with pre-hepatic portal hypertension following portal vein ligation.^37^ In the group with cirrhosis, HPS developed and the plasma levels of ET-1 and TNF-a increased. In the group with portal vein ligation, the rise in the plasma levels of TNF-a and HPS only occurred after infusion of exogenous ET-1*,* regardless of whether portal hypertension was present. This finding has generated discussion on the interaction between ET-1 and TNF-a and how these substances act on the pulmonary microvasculature in experimentally induced HPS.^37^

In cases of liver cirrhosis, deterioration of the hepatocytes and the Kupffer cells that make up the hepatic mononuclear phagocyte system occurs. This system plays an important role in the removal of microorganisms from the portal circulation.^25^ Moreover, the functional efficiency of the intestinal mucous barrier declines as the drainage of the portal blood becomes more difficult, thereby causing edema in the mucosa and reductions in intestinal peristalsis.^23^ Reductions in bile secretion also occur, which favor massive bacterial growth in the intestinal lumen, particularly Gram-negative bacteria,^23^ thereby allowing penetration and circulation of microorganisms and endotoxins.^23^^,^^38^

To demonstrate the role of bacterial translocation in pulmonary oxidative stress, models for cirrhosis due to CBDL were compared with a control group. In the group with cirrhosis, abnormalities compatible with HPS were observed in the arterial gases, along with high levels of enzymes, which reflected the degree of lipoperoxidation in the lung homogenate of these animals. This reflected likely phagocytic action by the pulmonary macrophages, for combating bacterial translocation.^36^

With the same aim of identifying the influence of bacterial translocation on the development of pulmonary vascular abnormalities, a rat model for cirrhosis induced by administration of a solution containing alcohol and cholesterol, followed by a solution of lipopolysaccharides containing *Escherichia coli*, was analyzed*.* There was an increase in endotoxin levels in the plasma, and the number of Gram-negative colonies in the mesenteric lymph nodes was closely associated with increased plasma levels of ET-1, NO and TNF-a, as well as rises in NO and ET-1 levels in the lung homogenate. This suggests that this organ may also be a source of ET-1, along with the liver, or that intestinal endotoxemia may irritate the Kupffer cells that release TNF-a, thereby either directly or indirectly inducing the production of ET-1.^38^

In order to determine whether there is an altered response by the vascular ET-1 receptors in patients with cirrhosis, exogenous ET-1 was injected into the forearm artery in cirrhotic patients and controls. While there was vasoconstriction in the forearm of the control group, there was vasodilatation in the forearm of the cirrhotic patients. Thus, the different response to endothelin may contribute towards generalized vasodilatation in patients with cirrhosis.^41^

Experimental studies have demonstrated consistent results regarding the high production of carbon monoxide (CO) that is associated with IPVD. However, the role of CO in the development of HPS in humans has yet to be clarified, even with findings of higher CO levels in cirrhotic patients with HPS than in cirrhotic patients without HPS.^43^

Experimental findings regarding the roles of ET-1, TNF-a and intestinal endotoxemia in the development of IPVD in the pulmonary microvasculature may contribute towards understanding the physiopathology of HPS in humans and allow the use of new treatments in the future.^5^^,^^33^ It has recently been suggested that, in addition to the functional changes, structural remodeling of the pulmonary microvasculature may occur in patients with HPS. This notion is based on liver transplant follow-up studies, in which limitations to the capacity for CO diffusion persist even after resolving the disorder in the pulmonary ventilation/perfusion ratio.^34^^,^^35^ Further studies on this subject are needed.

## DIAGNOSING OF HPS

The anatomical substrate and main structural change stemming from HPS is pulmonary capillary dilatation.^5^ These dilatations are believed to hinder the diffusion of O_2_ molecules from the alveolus to the center of the pulmonary capillary,^1^ where they normally bind with hemoglobin (Hb) molecules, thereby forming oxyhemoglobin, which distributes O_2_ to the different tissues.^44^ Excessive blood flow due to portal hypertension also occurs in these dilated vessels, which leads to a reduction in the time available for O_2_ molecules to bind to Hb molecules and causes an increase in alveolar-arterial oxygen tension difference (PA-a,O_2_).^24^ The increase in this gradient, which is associated with failure of the pulmonary vasoconstriction mechanism due to failure of the ET-1 receptors to regulate the pulmonary vascular tonus, is believed to cause arterial hypoxemia.^5^^,^^9^

In more severe cases of HPS, angiogenesis and true pulmonary arteriovenous communication occur.^1^ There is also a poor response to the O_2_ supply, even when administered at high concentrations, whereas this response is quite satisfactory in relation to IPVD.^1^^,^^45^

Based on the concept of the syndrome, diagnosis can be achieved through complementary examinations to prove the presence of IPVD and gas exchange abnormalities in patients with liver disease.^5^ Symptoms such as dyspnea and platypnea are common in HPS, but are not pathognomonic and can be found in the early phase, whereas spider angioma, peripheral cyanosis and finger clubbing are found in more advanced stages.^5^^,^^46^ The combination of non-specific clinical criteria, associated with the lack of standardization in the diagnostic criteria for defining HPS and the lack of a gold standard confirmation test^47^ may lead to diagnostic errors,^7^ This also explains the wide range of prevalence.^9^^,^^11^ There are reports of occurrences of HPS in patients with cirrhosis, with rates ranging from 4%^48^ to 32%.^9^^,^^10^^,^^12^^,^^49^^,^^50^ The frequency among non-cirrhotic patients with portal hypertension has been found to be lower, ranging from 8% to 9.7%.^29^^,^^30^ Among cases of chronic hepatitis with no cirrhosis or portal hypertension, the occurrence rate is 1.1%.^31^ In 2004, a paper was published suggesting standardization for diagnosing HPS,^5^ and this has contributed towards future comparisons between different studies. According to this guideline, HPS should be diagnosed if patients present liver disease associated with the presence of IPVD and arterial gas exchange abnormalities (PA-a,O_2_ ≥ 15 mmHg or PaO_2_ < 80 mmHg).^5^

## ARTERIAL GAS ANALYSIS

Although arterial gas analysis should be performed in order to confirm a diagnosis of HPS, a number of studies on the role of measurements of peripheral oxygen saturation using pulse oximetry (SpO_2_) for screening patients with liver cirrhosis have demonstrated that this quick, non-invasive test is useful for assessing orthodeoxia^51^ and for detecting hypoxemia in liver transplant candidates.^52^ SpO_2_ is also considered economically viable, with a better cost-benefit ratio than shown by administration of questionnaires on dyspnea. The use of this test has also been associated with improvement in the survival rate among liver transplant candidates, compared with lack of any screening procedure for HPS among such patients.^53^ However, it should be stressed that, through overestimating arterial oxygenation, SpO_2_ performed as a screening test may fail to identify milder cases of HPS that only exhibit a rise in PA-a,O_2_, without any hypoxemia yet.^3^

Although orthodeoxia has also been associated with HPS, its physiopathology remains insufficiently clarified.^5^^,^^50^ Cutoff points have yet to be validated for its use as a diagnostic criterion. However, it has been proposed that orthodeoxia is characterized by a reduction in PaO_2_ greater than 5% or at levels greater than 4 mmHg.^54^ Moreover, the physiopathology of hyperventilation and hypocapnia (PaCO_2_ < 35 mmHg), which is frequently found in patients with cirrhosis, is not yet completely understood.^55^^,^^56^ Nonetheless, it has been demonstrated that high levels of progesterone may be related to these abnormalities.^57^

The majority of studies suggest that arterial gas analysis is essential, beginning at the first consultation for cirrhotic patients who are candidates for liver transplantation. Furthermore, arterial blood analysis is recommended as a screening test for investigating HPS, which should be pursued in cases with high PA-a,O_2_ or hypoxemia.^3^^,^^58^ PA-a,O_2,_ in which PaCO_2_ is included as a component, has proven to be more sensitive than PaO_2_ alone for diagnosing HPS.^5^^,^^9^ After the management guidelines for HPS were published, in which PA-a,O_2_ ≥ 15 mmHg in liver disease patients with IPVD was deemed sufficient for confirmation of HPS, it was suggested that PaO_2_ should no longer be a separate diagnostic criterion and should also become part of the classification of the syndrome, with prognostic significance.^5^^,^^59^ HPS may therefore be classified as mild in cases of PaO_2_ ≥ 80 mmHg, moderate in cases of PaO_2_ < 80 mmHg or ≥ 60 mmHg, severe in cases of PaO_2_ < 60 mmHg or ≥ 50 mmHg and very severe in cases of PaO_2_ < 50 mmHg.^5^

## DIAGNOSING OF IPVD

In normal individuals at rest, the diameter of the pulmonary capillaries can reach 15 µm and PA-a,O_2_ is considered normal up to 8 mmHg.^5^^,^^44^ During aerobic exercise, a physiological opening with anatomical arteriovenous communication is believed to contribute towards worse performance in pulmonary gas exchange.^60^ The presence of this physiological intrapulmonary shunt has been observed through contrast echocardiography performed during exercise tests whenever arterial gas analysis reveals PA-a,O_2_ greater than 12 mmHg.^60^

In patients with HPS, IPVD occurs even at rest. It may reach as much as 500 µm in diameter and is located near gas exchange units, predominately in the lower lung fields, where the gravitational effect results in increased blood flow.^61^ Less frequently, these pulmonary vascular abnormalities are true arteriovenous communications.^5^

In the standardized diagnosis, two methods may be used to confirm the presence of IPVD: contrast echocardiography and lung perfusion scintigraphy using macroaggregated albumin labeled with technetium-99m (^99m^TcMAA).^5^ Scintigraphy allows quantification of the degree of IPVD, based on greater extrapulmonary uptake of the macroaggregates,^62^ which have a diameter greater than 20 µm and surpassed only by the dilated pulmonary capillaries.^63^^,64^

High-resolution computed tomography of the thorax is a recent imaging method for diagnosing IPVD. It has revealed that dilatations in peripheral lung vessels may have a good correlation with the severity of gas exchange abnormalities in patients with HPS. This method also allows simultaneous evaluation of pulmonary parenchyma in order to exclude other causes of hypoxemia.^65^^,^^66^^,^^67^^,^^68^ However, current diagnostic guidelines consider that its precision for diagnosing HPS has not yet been well established.^5^

Contrast echocardiography is the preferred method for diagnosing IPVD because of its greater sensitivity, in comparison with ^99m^TcMAA, and its ability to rule out intra-heart communication,^62^ which is responsible for false-positive results.^69^ Images obtained using this method are considered to be suggestive of IPVD when the left atrium is contrasted between the fourth and sixth cycles following opacification of the right atrium, in the absence of intra-heart communication. Such communication is considered present if the contrast reaches the left atrium by the third cycle.^64^^,^^69^^,^^70^ In normal situations, the contrast particles, which vary in diameter depending on the type of contrast used, are impacted in pulmonary capillaries of normal diameter. They are then physiologically absorbed by the alveoli and do not appear in the left atrium.^5^

There is discussion in the literature regarding whether this method, in the form of transthoracic echocardiography (TTE) should be the examination of choice for diagnosing IPVD, compared with transesophageal echocardiography (TEE).^69^ Different results have been obtained in studies using these methods on cirrhotic candidates for liver transplantation. While some authors stress the superiority of TEE, which avoids the false positives encountered in TTE,^70^^,^^71^ one study has stressed the greater efficacy and sensitivity of TTE over TEE, suggesting that the latter should used only in cases of negative TTE.^72^ However, these differences are probably due to differences in image quality, which is better when images are taken at the second harmonic frequency rather than at the fundamental frequency.^73^^,^^74^ There is one report in which higher occurrence of IPVD in comparison to findings from other studies^72^^,^^73^^,^^74^^,^^75^ was attributed to better resolution of the TTE images at the second harmonic.^76^

The sensitivity of TTE for diagnosing IPVD is currently questioned less than its capacity to rule out false-positive results, such as cases of patent foramen ovale.^69^^,^^75^ TTE studies have demonstrated greater sensitivity for diagnosing patent foramen ovale when the Valsalva maneuver is used to increase the pressure in the right atrium, thereby facilitating the opening of this orifice and reducing the number of false-positive findings of IPVD.^77^

Although echocardiography is better than ^99m^TcMAA, there may be false-positive results with the TTE technique, depending on the echocardiographic technique used.^72^^,^^74^ In fact, studies that have evaluated the TTE technique, taking the TEE as the gold standard, have described sensitivity of 68% to 75% and specificity of 93% to 100% for TTE.^72^^,^^74^ In addition, there is evidence in the literature that TTE with contrast is as accurate as TEE with contrast for determining the presence of right-to-left shunt. However, further studies are needed to describe the equivalence between these two techniques, in order to determine the source of the right-to-left shunt.^69^

The volume of the left atrium and degree of right ventricle dysfunction determined through echocardiography have recently been suggested as possible parameters for diagnosing HPS in patients with cirrhosis,^49^^,^^78^ but so far, there has not been any standardization of the diagnostic guidelines.

## DIAGNOSING OF LUNG DISEASE

Excluding patients with chronic lung disease as part of the differential diagnosis for HPS is no longer necessary, as there are a number of better established criteria for this syndrome.^5^^,^^79^ However, investigation of associated lung disease is recommended among patients with very severe HPS, in order to determine whether the transplant protocol is appropriate. Such cases should be analyzed individually,^5^ because the presence of advanced lung disease is an absolute contraindication for liver transplantation.^16^

However, along with routine investigation of HPS among liver transplant candidates with suspected chronic lung disease, a preoperative lung evaluation should be performed regularly.^5^ This evaluation seeks to calculate the potential risk of postoperative pulmonary complications through specific clinical examination, spirometry and chest x-ray.^80^ A chest x-ray may reveal underlying parenchymatous diseases or bilateral basal interstitial opacities. These may be related to IPVD, but their presence is not a criterion of sufficient sensitivity for them to be considered adequate for diagnosing IPVD.^1^^,^^79^^,^^81^ The CO diffusion test, ^99m^TcMAA and high-resolution computed tomography may be performed based on individual evaluations on each patient.^5^

## HPS TREATMENT AND PROGNOSIS

With no clear definition of the pathogenesis of HPS, there is as yet no treatment that offers satisfactory results for its reversion.^6^^,^^82^ Treatment options such as inhaled N^G^-nitro-L-arginine methyl ester (L-NAME),^35^ intravenous methylene blue,^83^ somatostatin,^84^ almitrine, indomethacin, norfloxacin, etc., have been tested on HPS cases, but there is a lack of clinical trials to support their use.^4^^,^^7^

The invasive procedure of transjugular intrahepatic portosystemic shunt has not resulted in success when used for treating HPS, although it is widely used for treating cirrhotic patients with refractory ascites and digestive bleeding relating to portal hypertension.^82^ On the other hand, cavoplasty, which is cited as a treatment option for patients with Budd-Chiari syndrome, has resulted in complete reversion of HPS.^85^ Embolization of possible arteriovenous communications has also been reported to have been successful.^86^ Oxygen therapy is indicated to improve the clinical condition of patients with severe hypoxemia who are known to be responsive to O_2_ supplementation.^45^ This therapy thus makes it possible for such patients to wait for transplant surgery.^5^

When diagnoses of HPS have been established, the aim is for this to have a positive influence on the prognosis for liver transplant candidates.^5^ No treatment tested so far has proven capable of altering the natural course of HPS,^35^ except liver transplant surgery.^61^^,^^87^^,^^88^^,^^89^ It should be stressed that this is a recent indication, since HPS was previously considered to be a contraindication for liver transplant surgery.^64^

Because HPS may be an independent risk factor for worse prognosis among liver transplant candidates,^14^^,^^59^ there is a current formal recommendation to prioritize transplantation for patients with the severe form of this syndrome.^18^^,^^22^ However, each HPS case that is considered severe should be analyzed individually because of the high morbidity and mortality rates associated with this condition, both during surgery and during the postoperative period.^5^^,^^14^^,^^47^

Without liver transplantation, the long-term mortality rate is higher among cirrhotic patients with HPS than among those without HPS.^13^^,^^90^ Prospective analysis among candidates for liver transplantation has demonstrated significantly lower mean survival among patients with HPS than among those without HPS (4.8 months and 35.3 months respectively; P = 0.005) when they do not receive a transplant, even compared with the same classification of Child-Pugh score (0.26 months and 3.82 months respectively; P = 0.01).^90^

With the objective of evaluating the impact of liver transplantation on the survival of patients with HPS, a case-control study compared patients with HPS who did and did not undergo liver transplantation. The mortality rates observed were 21% and 78%, respectively. In the same study, comparing patients with and without HPS who did not receive a transplant, the mean survival was 24 months for the group with HPS and 87 months for the group without HPS, with five-year survival of 23% and 63%, respectively (P = 0.0003).^13^ This is likely to have been due to the rapid progression of hypoxemia in the patients with HPS and the mean annual decline in PaO_2_ of approximately 5 mmHg.^13^

It has been shown that, following transplantation, the oxygen saturation improves, the oxygen dependence of HPS patients is resolved^47^ and there are similar mortality rates among patients with and without HPS.^13^ These findings confirm the changes to the course of HPS and consequent prognosis for such patients that transplantation causes.^5^

A number of studies have sought to correlate the severity of liver dysfunction, as classified by the Child-Pugh score, with the severity of hypoxemia, but no statistical significance has been found for such an association.^14^^,^^55^^,^^63^^,^^91^^,^^92^ Nonetheless, there has been reference to an association between hypoxemia and liver cirrhosis.^56^ It remains uncertain whether any association exists between severity of liver dysfunction and occurrences of HPS, considering that there are reports both against^10^^,^^11^^,^^51^ and in favor of such an association.^9^^,^^90^^,^^93^^,^^94^

The decision to give priority to patients with HPS, among the candidates for liver transplant surgery, has been questioned based on the lack of complete understanding of its etiopathogenesis and physiopathology, as well as on the lack of a single test to confirm the diagnosis of this syndrome.^7^ Giving priority to patients with HPS because they are considered to be more serious cases may not reduce these patients’ mortality rate, but rather, it may transfer mortality to the postoperative period. Meanwhile, transplantation for cases of lesser severity is postponed: in principle, such patients have a better prognosis, but will be subject to longer waits before receiving the transplant.^95^ This may lead to future questions involving HPS among liver transplant candidates, with regard to the recent determinations of government institutions that administrate organ distribution.

It is hoped that future studies will provide complete understanding of the physiopathology of this syndrome, thereby ensuring its precise diagnosis and pharmacological treatment. Such treatment may one day be as effective as liver transplantation, which today changes the natural course of HPS and provides a greater survival rate.

The recognized influence of HPS on the prognosis of candidates for liver transplantation justifies routine, standardized diagnostic investigation of HPS in referral hospitals. Meanwhile, cirrhotic patients with severe HPS are prioritized on the waiting list for liver transplantation and those with very severe HPS should be evaluated individually due to the high surgical risks. Cirrhotic patients with mild to moderate HPS should be evaluated periodically until new studies redirect the treatment protocol.
